# Lysophosphatidylethanolamine Affects Lipid Accumulation and Metabolism in a Human Liver-Derived Cell Line

**DOI:** 10.3390/nu14030579

**Published:** 2022-01-28

**Authors:** Yusuke Yamamoto, Toshihiro Sakurai, Zhen Chen, Nao Inoue, Hitoshi Chiba, Shu-Ping Hui

**Affiliations:** 1 Faculty of Health Sciences, Hokkaido University, Kita-12, Nishi-5, Kita-ku, Sapporo 060-0812, Japan; yammasxy@gmail.com (Y.Y.); sakura@hs.hokudai.ac.jp (T.S.); chenzhen@hs.hokudai.ac.jp (Z.C.); naobee0628@eis.hokudai.ac.jp (N.I.); 2 Department of Nutrition, Sapporo University of Health Sciences, Nakanuma Nishi-4-2-1-15, Higashi-ku, Sapporo 070-0894, Japan; chiba-h@sapporo-hokeniryou-u.ac.jp

**Keywords:** lipid droplets, LC-MS/MS, triacylglycerol, adipose tissue triglyceride lipase, catabolism

## Abstract

The physiological functions of lysophosphatidylethanolamine (lysoPE) have not been fully elucidated. In this study, the effects of lysoPE on lipogenesis and lipolysis were investigated in a cultured human liver-derived cell line. The intracellular lipid profile was investigated in detail using liquid chromatography–tandem mass spectrometry (LC-MS/MS) to better understand the underlying mechanism. The expression of genes related to lipid metabolism and catabolism was analyzed using real-time PCR. LysoPE supplementation induced cellular lipid droplet formation and altered triacylglycerol (TAG) profiles. Furthermore, lysoPE downregulated expression of the TAG hydrolyzation regulation factor *ATGL*, and reduced the expression of fatty acid biosynthesis-related genes *SREBP1* and *SCD1*. LC-MS/MS-based lipidomic profiling revealed that the addition of lysoPE 18:2 increased the PE species containing linoleic acyl, as well as the CE 18:2 species, likely due to the incorporation of linoleic acyl from lysoPE 18:2. Collectively, these findings suggest that lysoPE 18:2 is involved in lipid droplet formation by suppressing lipolysis and fatty acid biosynthesis. Thus, lysoPE might play a pathological role in the induction of fatty liver disease.

## 1. Introduction

Lysophosphatidylethanolamine (lysoPE) is a lysophospholipid that is a deacylated product of phosphatidylethanolamine (PE) hydrolysis induced by phospholipase A1/A2 [1]. LysoPE is a minor component of the cell membrane [2] and stimulates chemotactic migration and infiltration of ovarian cancer cells [3]. LysoPE also functions as a neuronutrient activator through the mitogen-activated protein kinase signaling pathway in pheochromocytoma cells [4]. LysoPE was recently reported to be involved in the stimulation of neurite outgrowth and protection against glutamate toxicity in cultured cortical neurons [5,6]. A previous study showed that lysoPE inhibits lipopolysaccharide-induced M1 macrophage polarization in mouse peritoneal macrophages [7]. However, the function of lysoPE in the liver has not been reported. Lysophosphatidylcholine (lysoPC) is structurally similar to lysoPE but differs in its headgroup (choline instead of ethanolamine) and is known to form lipid droplets in endothelial cells [8]. However, whether lysoPE has a similar effect on liver steatosis remains unknown.

The incidence of non-alcoholic fatty liver disease (NAFLD) is continuously increasing worldwide, with a prevalence of 20–30% in Europe, the Middle East and Japan, and is estimated to be 33% in the United States [9]. NAFLD is defined as a multifactorial metabolic disease composed of simple steatosis, or non-alcoholic fatty liver (NAFL), and non-alcoholic steatohepatitis (NASH). Simple steatosis can progress to NASH, cirrhosis and hepatocellular carcinoma owing to multiple factors, including oxidative stress and insulin resistance [10]. Lipidomic studies revealed an increase in lipid hydroperoxides in both in vitro (lipid droplets from fatty-acid-supplemented hepatocytes) and in vivo (liver of diet-induced mice) studies [11,12]. Therefore, fatty liver may be a potential risk factor for NASH progression and should be prevented. Fructose and free fatty acids such as palmitic acid, oleic acid and linoleic acid are known to induce hepatic fat accumulation, and can be applied to cells or animals as hepatic steatosis models [11,13]. It is important to understand whether lysoPE is involved in the alteration of lipid metabolism, which includes hepatic lipid accumulation; however, the physiological functions of each lysoPE molecular species remain unknown. Their elucidation may lead to a discovery of a novel pharmacological target for hepatic steatosis as well as new physiological functions of lysoPE.

A major challenge in elucidating the physiological functions of lysoPE is the unavailability of individual lysoPE molecular species. In our previous study, a series of authentic lysoPE molecular species was prepared via chemical synthesis [14,15]. LysoPE 18:2 was the most abundant species in the serum of healthy subjects, as determined by using liquid chromatography–tandem mass spectrometry (LC-MS/MS) analysis [16].

Therefore, the aims of our study were (1) to investigate the physiological functions of lysoPE, targeting its effect on lipid accumulation in human hepatocytes; (2) to explore the modulation of intracellular lipid metabolism, including the changes in lipid content and the alterations of molecular composition and (3) to analyze the expression of the genes related to lipid metabolism and catabolism. The graphical representation of the present study workflow is shown in the supplementary data (Scheme S1). Here, a combined analysis of lipidomic profiling, biochemical examination and gene expression assays were performed to better understand the biological effects of lysoPE.

## 2. Materials and Methods

### 2.1. Cell Culture

The human liver-derived cell line C3A (CRL-10741; ATCC, Manassas, VA, USA) was incubated in minimum essential medium (MEM; Thermo Fisher Scientific, Waltham, MA, USA) with GlutaMAX containing 10% fetal bovine serum (FBS; Biosera, Kansas City, MO, USA) and penicillin-streptomycin-neomycin (Thermo Fisher Scientific) at 37 °C in a 5% CO_2_ atmosphere prior to experimentation.

### 2.2. Cell Viability Assay

To assess cell viability, a 100 µL aliquot of a suspension of C3A cells was seeded in 96-well plates (2.0 × 10^4^ cells/well). After 24 h, the medium was replaced with 100 µL MEM containing 1% fatty-acid-free bovine serum albumin (BSA), without or with lysoPE 18:2 which was previously synthesized [15], and incubated at 37 °C in a 5% CO_2_ atmosphere for 24 h. WST-1 was added at a concentration of 5 µL/well three hours before incubation was ended. The absorbance was measured at 450 nm using a plate reader (ARVO-MX; PerkinElmer, Waltham, MA, USA). Results are expressed as the percentage absorbance compared to the control, which was set to 100%.

### 2.3. Oil Red O Staining

A suspension of C3A cells (1 mL) was seeded in 24-well plates at a concentration of 2.0 × 10^5^ cells/well. After 24 h, the medium was replaced with 1 mL MEM containing 1% fatty-acid-free BSA, without (control group) or with lysoPE 18:2 (lysoPE 18:2-supplemented group; *n* = 4, each group) and incubated at 37 °C in a 5% CO_2_ atmosphere for another 24 h. The supernatant was removed, and each well was washed twice with phosphate-buffered saline (PBS). The cells were then fixed by incubation in 200 µL of 10% neutral-buffered formalin solution for 10 min. The cells were washed twice with 200 µL PBS and isopropanol (60%) was added (200 µL), followed by incubation for 10 min. The isopropanol was removed and 200 µL of Oil Red O solution was added, followed by incubation for 20 min to stain the cells. Each well was washed with 60% isopropanol and then 200 µL of PBS was added. The cells were observed and images were obtained using a microscope (IX71; Olympus, Tokyo, Japan). The Oil Red O dye was then extracted using isopropanol and the extracts were transferred to another 96-well plate. Isopropanol was used as the blank. The absorbance of the Oil Red O extract was measured at 550 nm using a plate reader (xMark Microplate Spectrophotometer; Bio-Rad, Hercules, CA, USA). The control was set to 100%.

### 2.4. Lipidomic Analysis Using Orbitrap LC-MS/MS

C3A cells (1 mL) were seeded in 24-well plates at a concentration of 2.0 × 10^5^ cells/well and incubated for 24 h. The medium was then replaced with 1 mL of MEM containing 1% fatty-acid-free BSA, without or with 20 µM lysoPE 18:2, and incubated at 37 °C in a 5% CO_2_ atmosphere for 24 h. The cells were washed with PBS and collected in 100 µL of radioimmunoprecipitation assay (RIPA) buffer (FujiFilm Wako Pure Chemical, Tokyo, Japan) containing a protease inhibitor cocktail (Sigma-Aldrich, St. Louis, MO, USA) and phenylmethylsulfonyl fluoride (G-Biosciences, St. Louis, MO, USA) (98:1:1). Cell lysates from four duplicate wells (under the same conditions) were mixed (400 µL) to obtain a sufficient volume for the assay. Six samples were prepared for each experimental group. A 50 µL aliquot of each pooled cell lysate was used to measure the protein concentration using the bicinchoninic acid (BCA) assay. The remaining 350 µL was used for comprehensive lipid analysis via Orbitrap LC-MS/MS. The lipid classes evaluated were triacylglycerol (TAG), cholesteryl ester (CE), PE, lysoPE, PC and lysoPC. TAG and CE were analyzed as the core lipids in intracellular lipid droplets. Each lipid species amount was normalized to the amount of protein, as determined using the BCA assay, which was measured using a commercial kit (Thermo Fisher Scientific).

The Orbitrap LC-MS/MS conditions were previously described [17]. An Atlantis T3 C18 column (2.1 mm × 150 mm, 3 μm; Waters, Milford, MA, USA) was used for chromatographic separation. The mobile phase consisted of a 10 mM ammonium acetate aqueous solution (A), isopropanol (B) and methanol (C). The measurements were obtained using optimized gradient elution in electrospray ionization positive and negative modes, with *m/z* ranges set at 150–1100 and 220–1650, respectively. MS/MS analysis was performed via collision-induced dissociation in ion trap mode. Raw data were processed using Xcalibur 2.2 (Thermo Fisher Scientific). The mass tolerance was within 5.0 ppm, and the acyl composition was identified by comparing the MS/MS fragments with the LIPIDMAPS database [18].

### 2.5. RNA Extraction and cDNA Conversion

C3A cells were seeded in 24-well plates at a concentration of 2.0 × 10^5^ cells/well and incubated for 24 h. The medium was then replaced with 1 mL of MEM containing 1% fatty-acid-free BSA, without or with lysoPE 18:2 (20 µM), and incubated at 37 °C in a 5% CO_2_ atmosphere for 24 h. The cells were washed with phosphate-buffered saline (PBS), followed by RNA extraction using the PureLink RNA Mini Kit (Thermo Fisher Scientific), according to the manufacturer’s protocol. RNA concentrations and purity were measured using a NanoDrop spectrophotometer (Thermo Fisher Scientific). Next, 500 ng of RNA was converted to complementary DNA (cDNA) using the ReverTra Ace qPCR RT Master Mix with gDNA Remover (Toyobo, Osaka, Japan), according to the manufacturer’s protocol. The acquired cDNA was stored at −80 °C until use.

### 2.6. Gene Expression Analysis Using Real-Time PCR

Gene expression of adipose tissue triglyceride lipase (*ATGL*), diacylglycerol acyltransferase 1 (*DGAT1*), sterol regulatory sequence binding protein 1 (*SREBP1*), stearoyl CoA desaturase 1 (*SCD1*), peroxisome proliferator-activated receptor γ (*PPARγ*), *CD36* and glyceraldehyde-3-phosphate dehydrogenase (*GAPDH*) was analyzed through real-time PCR using the THUNDERBIRD SYBR qPCR Mix (Toyobo), according to the manufacturer’s protocol. Primer sequences are listed in Table S1 [19,20,21,22]. A CFX Connect Real-Time System real-time PCR analysis system (Bio-Rad Laboratories, Hercules, CA, USA) was used for all assays. All results were normalized to the expression level of the housekeeping gene, *GAPDH*.

### 2.7. Statistical Analysis

All statistical analyses were performed using GraphPad Prism 7.0e (GraphPad Software, La Jolla, CA, USA). For comparisons of three or more groups, one-way analysis of variance (ANOVA), followed by Dunnett’s test, was applied. The *t*-test was used for the comprehensive analysis of lipids using Orbitrap LC-MS/MS and real-time PCR. Statistical significance was set at *p* < 0.05. All results are depicted as the mean ± standard deviation.

## 3. Results

### 3.1. Supplementation of LysoPE 18:2 Showed No Cytotoxicity to Hepatocyte Cell Line

**Figure 1:**
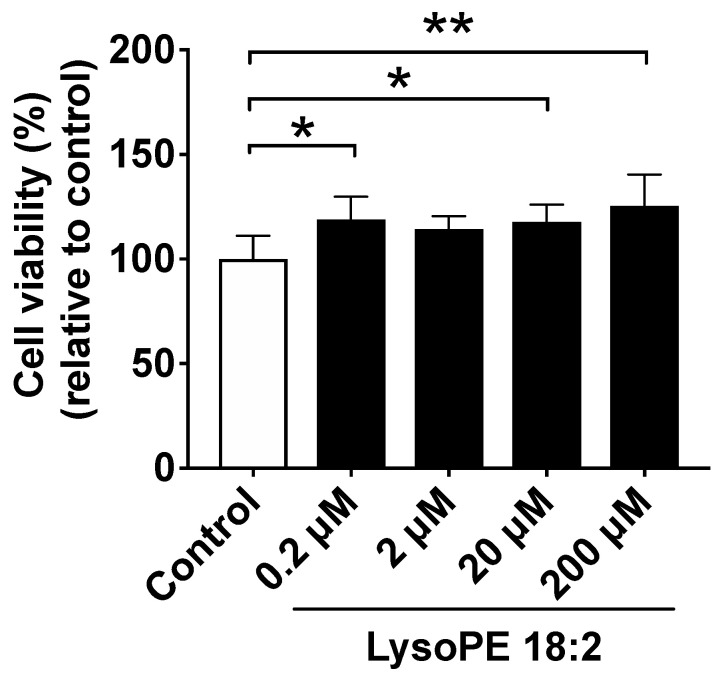
Cell viability assay of C3A liver-derived cells at 24 h after adding lysoPE 18:2. Cell viability in the control group was set as 100%. Results are expressed as mean ± standard deviation. *n* = 6. * *p* < 0.05, ** *p* < 0.01.

To confirm the cell toxicity of lysoPE 18:2 and to determine the appropriate concentration of lysoPE 18:2 for C3A cells, we initially tested the cell viability within the range up to 200 µM. As a result, cells treated with lysoPE 18:2 at the tested concentrations of 0.2 µM, 2 µM, 20 µM and 200 µM showed 118.8% ± 11.0%, 114.4% ± 6.1%, 117.7% ± 8.3% and 125.3% ± 15.0% of the cell viability compared with control, respectively (Figure 1). These data suggest that lysoPE 18:2 did not cause cytotoxicity to C3A cells. Moreover, although the cell viability was even slightly increased with the addition of lysoPE 18:2 at 0.2, 20 and 200 µM, there was no evidence showing a dose-dependent effect on hepatocyte proliferation. Thus, there was no cell toxicity induced by lysoPE 18:2 up to a concentration of 200 µM.

### 3.2. LysoPE 18:2 Supplementation Increased the Number of Lipid Droplets in Hepatocytes

**Figure 2:**
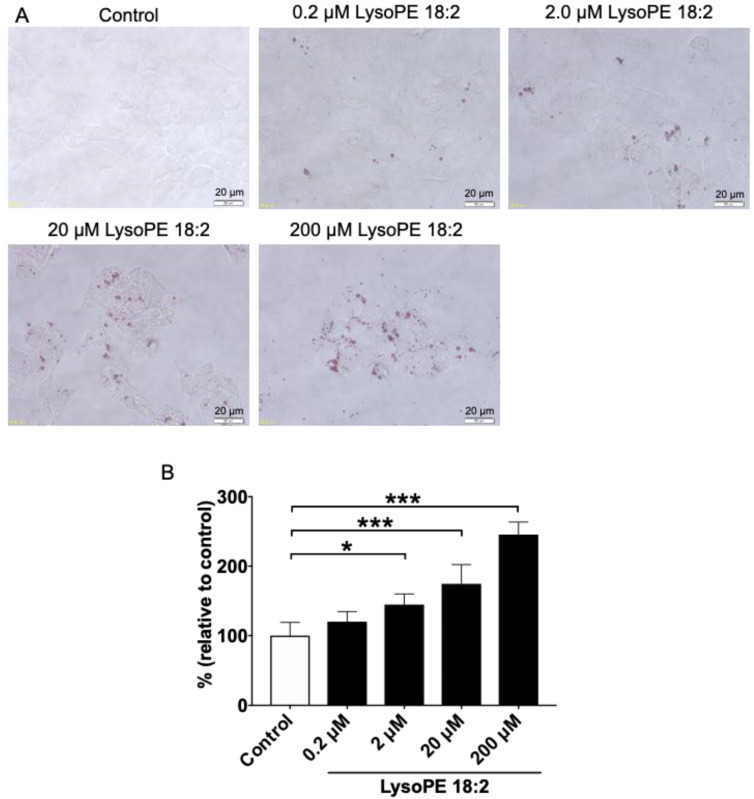
Oil Red O staining and evaluation of lipid accumulation. (**A**) Lipid droplets formed by C3A cells supplemented with 0.2–200 µM lysoPE 18:2 (40×). (**B**) Quantification of lipid accumulation, as measured by the absorbance of extracted Oil Red O dye. Absorbance in the control group was set as 100%. Results were expressed as mean ± standard deviation. *n* = 4. * *p* < 0.05, *** *p* < 0.001.

Lipid staining with Oil Red O was performed to observe morphological features after lysoPE 18:2 treatment, especially lipid droplets. More lipid droplets, which were dyed red, were observed in cells supplemented with 0.2–200 µM lysoPE 18:2. The increase was also dose-dependent (Figure 2A). The absorbance of Oil Red O was measured to express the degree of lipid droplet formation. The absorbance was significantly elevated in a dose-dependent manner in cells supplemented with 2–200 µM lysoPE 18:2 (Figure 2B), consistent with microscopic observations.

### 3.3. Hepatic Lipid Profile Changed following LysoPE 18:2 Supplementation

**Figure 3:**
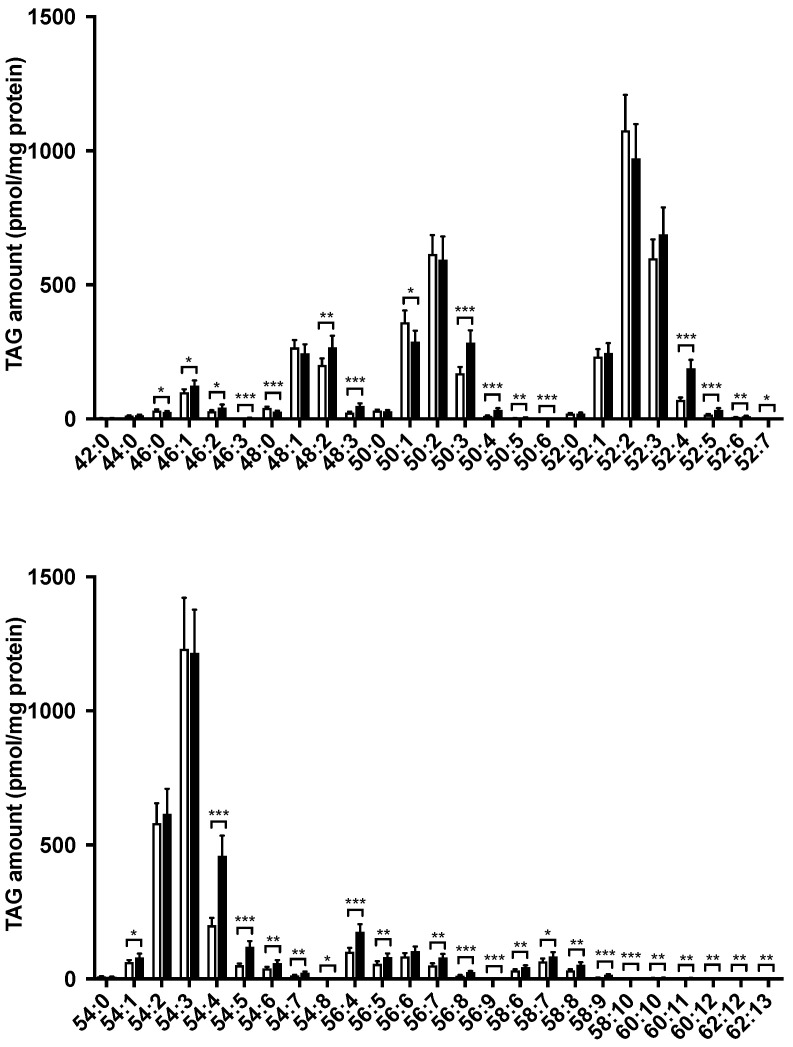
Comparison of TAG species in the lysoPE-supplemented (black bars) and unsupplemented (white bars) C3A cells detected using Orbitrap LC-MS/MS. Results are shown as mean ± standard deviation. *n* = 6. Student’s *t*-test, * *p* < 0.05, ** *p* < 0.01, *** *p* < 0.001.

To better understand the biological effects of lysoPE, we investigated intracellular lipid profiles using LC-MS/MS. TAG and CE species are the major components of lipid droplets. We detected 51 molecular TAG species (Figure 3), 33 of which were significantly increased in the lysoPE 18:2-supplemented group. Notably, 14 species showed a more than 2-fold increase. TAG 54:4, which increased 2-fold in the lysoPE 18:2-supplemented group, was identified as linoleic acyl using MS/MS analysis. Conversely, the levels of three molecular species, TAG 46:0, 48:0 and 50:1, were significantly decreased in the lysoPE 18:2-supplemented group.

**Figure 4:**
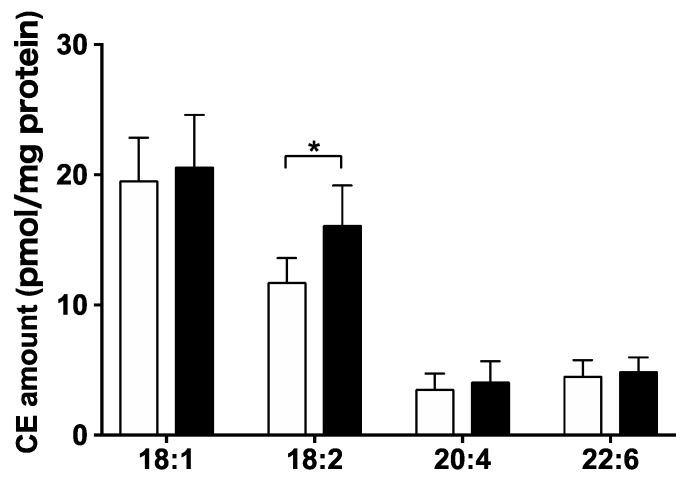
Comparison of CE species in lysoPE-supplemented (black bars) and unsupplemented (white bars) C3A cells detected using Orbitrap LC-MS/MS. Results are shown as mean ± standard deviation. *n* = 6. Student’s *t*-test, * *p* < 0.05.

Four molecular CE species were also detected. CE 18:2 was significantly increased in the lysoPE 18:2-supplemented group (*p* < 0.05). However, there were no obvious differences in other CE species among the groups (Figure 4).

**Figure 5:**
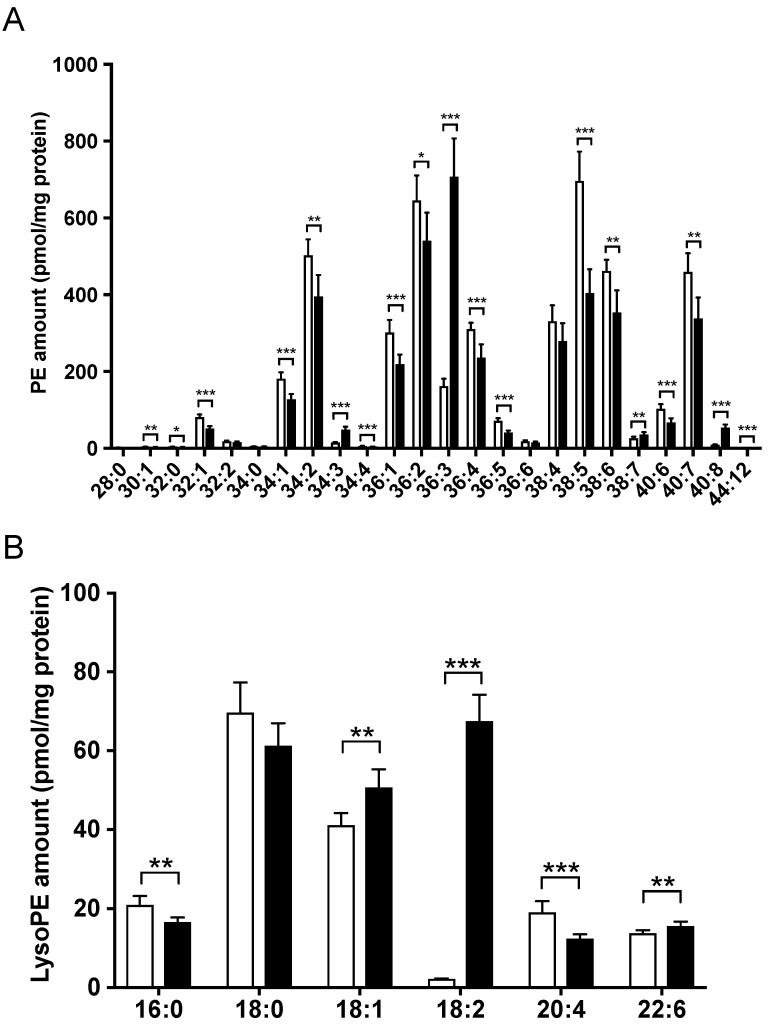
Comparison of PE (**A**) and lysoPE (**B**) species in lysoPE-supplemented (black bars) and unsupplemented (white bars) C3A cells detected using Orbitrap LC-MS/MS. Results are shown as mean ± standard deviation. *n* = 6. Student’s *t*-test, * *p* < 0.05, ** *p* < 0.01, *** *p* < 0.001.

We also analyzed PE and lysoPE to investigate the dynamics of these molecular species. Subsequently, 24 molecular PE species were detected. There were significant increases in levels of PE 34:3, 36:3, 38:7, 40:8 and 44:12 in the lysoPE 18:2-supplemented group, compared to those in the control group (Figure 5A). According to the MS/MS fragments, PE 36:3 and 40:8 contained linoleic acyl. All other molecular species were significantly decreased in the lysoPE 18:2-supplemented group.

Six molecular lysoPE species were detected. LysoPE 18:2 was the most abundant species in the lysoPE 18:2-supplemented group (Figure 5B). It should be noted that this included the supplemented lysoPE 18:2. LysoPE 18:1 and 22:6 levels were also significantly increased, whereas lysoPE 16:0 and 20:4 were significantly decreased in the lysoPE 18:2-supplemented group.

**Figure 6:**
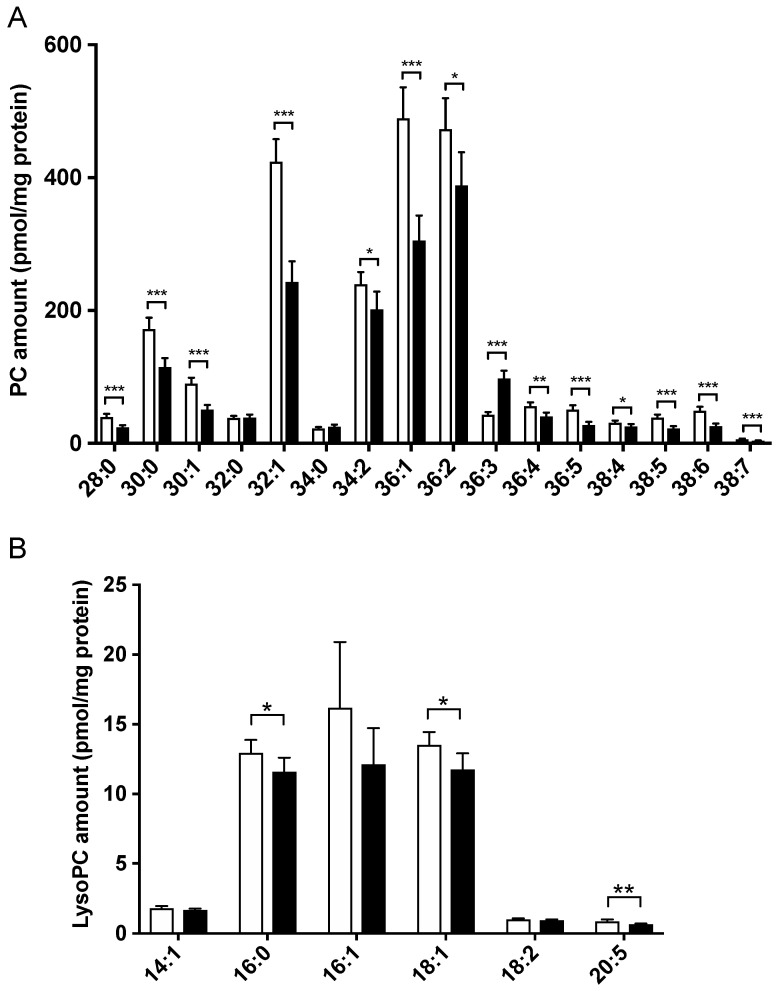
Comparison of PC (**A**) and lysoPC (**B**) species in lysoPE-supplemented (black bars) and unsupplemented (white bars) C3A cells detected using Orbitrap LC-MS/MS. Results are shown as mean ± standard deviation. *n* = 6. Student’s *t*-test, * *p* < 0.05, ** *p* < 0.01, *** *p* < 0.001.

To investigate the behavior of the major cellular phospholipids, we analyzed the PC profile and identified 16 molecular species. Overall, PCs were lower in the lysoPE 18:2-supplemented group (Figure 6A). Levels of PC 28:0, 30:0, 30:1, 32:1, 34:2, 36:1, 36:2, 36:4, 36:5, 38:4, 38:5, 38:6 and 38:7 were significantly decreased in the lysoPE 18:2-supplemented group compared with those in the control group. Only PC 36:3 was increased in supplemented cells, and was identified using MS/MS analysis as PC 18:1/18:2, a linoleic acyl-containing molecule.

We further analyzed the lysoPC species as the major lysophospholipids to better understand the changes in lysoPE 18:2 supplementation. Six molecular lysoPC species were identified. LysoPC species were also reduced in the lysoPE 18:2-supplemented group (Figure 6B), with a significant decrease in lysoPC 16:0, 18:1 and 20:5.

### 3.4. LysoPE 18:2 Supplementation Altered the Expression of Genes Related to Lipid Metabolism

**Figure 7:**
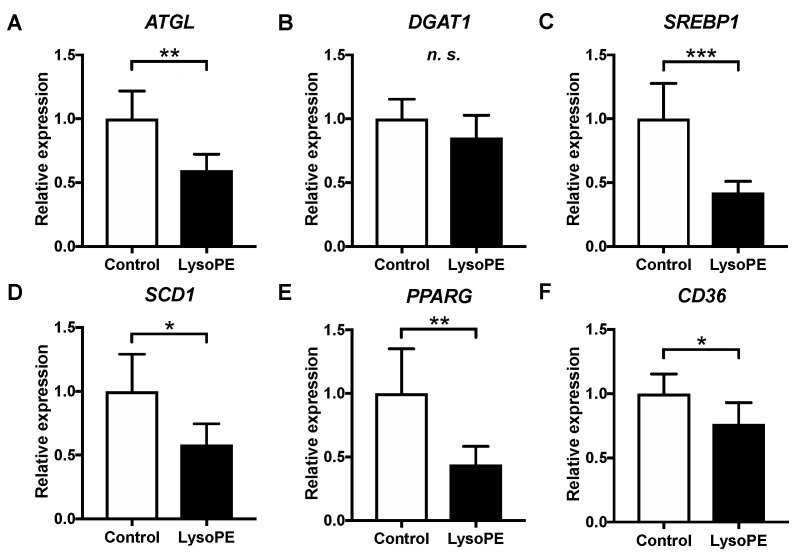
Expression of genes related to lipid droplet formation in lysoPE 18:2-supplemented and unsupplemented C3A cells. (**A**) *ATGL*; (**B**) *DGAT1*; (**C**) *SREBP1*; (**D**) *SCD1*; (**E**) *PPARG* (*PPARγ*); (**F**) *CD36*. The vertical axis shows the relative expression levels compared to the untreated control cells, which was set to 1.0. *n* = 6. Student’s *t*-test, * *p* < 0.05, ** *p* < 0.01, *** *p* < 0.001, *n.s.* not significant.

To determine whether lysoPE 18:2 affects lipid metabolism at the transcriptional level, we analyzed the expression of several genes involved in TAG catabolism (*ATGL*), TAG synthesis (*DGAT1*), de novo fatty acid synthesis (*SREBP1* and *SCD1*) and FA uptake (*PPARγ* and *CD36*) using real-time PCR. *ATGL*, *SREBP1* and *SCD1* expression was significantly lower in the lysoPE 18:2-supplemented group than in the control group (Figure 7). In contrast, *DGAT1* levels did not differ between the two groups.

## 4. Discussion

We found that lysoPE supplementation induced lipid droplet formation in hepatocytes in a dose-dependent manner, similar to a previous report demonstrating that lysoPC formed lipid droplets in endothelial cells [8]. This suggests that lysoPE may induce lipid droplet formation in hepatocytes and may be related to the pathophysiology of NAFLD. Levels of both serum lysoPE 18:2 and 20:4 were significantly decreased in mice with fatty livers fed a high-fat diet [23]. Studies have shown that serum lysoPE levels in both simple steatosis and NASH are lower than those in healthy subjects [15,24]. Hepatic lysoPE species were shown to be higher in patients with simple steatosis than in those with normal steatosis [25,26], whereas hepatic lysoPE levels were lower in NASH than in simple steatosis [25]. These findings indicate the promotion of lysoPE uptake from the plasma to the liver during simple steatosis. The specific receptor for lysoPE remains to be determined.

TAG and CE are the major lipid components in LDs [27]. Our lipidomic data showed that lysoPE supplementation induced an overall increase in TAG. In particular, TAG 54:4 increased 2-fold in the lysoPE 18:2-supplemented group. TAG 54:4 also contained linoleic acyl. This linoleic acyl may have originated from the linoleic acyl produced by the decomposition of lysoPE 18:2; this finding is supported by a previous study showing that the loading of fatty acids into cells induces TAG formation, including fatty acyl [28]. Similarly, CE with linoleic acyl was the only CE species that increased in the lysoPE 18:2-supplemented group, which may have originated from the linoleic acyl produced by the decomposition of lysoPE 18:2.

PE species that were markedly increased in the lysoPE 18:2-supplemented groups, such as PE 36:3 (18:1/18:2) and 40:8 (18:2/22:6), also contained linoleic acyl. The supplemented lysoPE 18:2 may have been a substrate for the increased production of these PE species. The cleavage of linoleic acyl from the increased PE 36:3 and 40:8 may be the source of the increase in lysoPE 18:1 and 22:6. In contrast, most PE and PC species were reduced in lysoPE 18:2-supplemented cells. PEs and PCs are mainly synthesized via the Kennedy pathway in the endoplasmic reticulum (ER) [29]. ER stress is present in NAFLD [30,31], which is followed by a decrease in hepatic PE. Thus, the overall PE reduction observed in the present study might be due to impaired PE production caused by ER stress. Although supplementation with lysoPE 18:2 markedly elevated lysoPE 18:2 levels, it did not significantly affect its profile. Additionally, the increase in lysoPE 18:2 levels reflected the addition of lysoPE 18:2 into the cells.

Changes in the expression levels of genes related to lipid metabolism and catabolism were also observed. *ATGL* expression was significantly downregulated in lysoPE 18:2-supplemented cells. ATGL hydrolyzes TAGs, the main component of lipid droplets, and converts them to diacylglycerols (DAGs) [32]. Conversely, DGAT1 is an enzyme that acylates DAGs and synthesizes TAGs [33]. Deficiency of hepatic ATGL in mice causes progressive hepatic steatosis [34,35], which suggests a suppression of TAG degradation in lipid droplets. Thus, a reduction in hepatic ATGL expression and activity could lead to the development of metabolic diseases, including NAFLD [36]. Mutations in the *ATGL* gene were observed in patients with hepatic steatosis and cardiac myopathy with systemic TAG accumulation in [37]. Supporting these reports, the present results suggest that lysoPE may induce TAG accumulation by downregulating *ATGL* expression.

**Figure 8:**
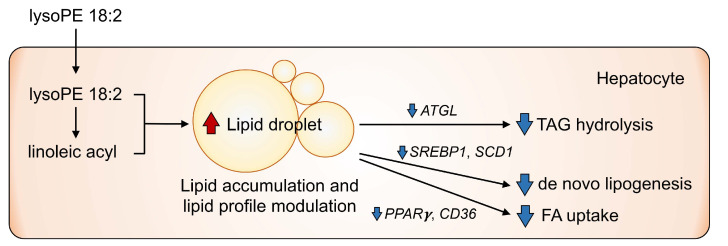
Putative effects of lysophosphatidylethanolamine (lysoPE) on hepatic lipid metabolism. The present study demonstrated that lysoPE 18:2 supplementation induced hepatic lipid accumulation and caused lipid profile alteration, possibly related to the suppression of lipolysis, fatty acid synthesis and fatty acid uptake.

More extensive lipid metabolism markers were analyzed to elucidate hepatic steatosis [38,39,40]. *SREBP1* and *SCD1* expression were significantly reduced in lysoPE 18:2-supplemented cells. *SCD1* catalyzes the biosynthesis of monovalent unsaturated fatty acids from saturated fatty acids [41]. SREBP1 is a transcription factor that regulates *SCD1* expression and controls fatty acid biosynthesis [42]. Additionally, hepatic *SCD1* levels are decreased in fatty liver model mice on a fed a high-fat diet [43,44]. *SCD1*-deficient mice are protected against steatosis owing to a reduction in lipogenesis and activation of β-oxidation [45,46]. The present results also showed that excessive lipid accumulation induced by lysoPE 18:2 could contribute to the decreased *SREBP1* and *SCD1* expression, which might be part of a feedback loop that suppresses the intracellular biosynthesis of excess fatty acids. Furthermore, *PPARγ*, a major regulator of lipid metabolism, controls FA uptake and de novo hepatic lipogenesis. Hepatic knockdown of *PPARγ* in mice leads to resistance to high-fat-diet-induced hepatic steatosis and the decrease of genes involved in lipogenesis (*SREBP1* and *SCD1*) and FA transporters regulated by *PPARγ* (*CD36*) [47]. Consistent with this, the downregulation of *PPARγ, CD36, SREBP1* and *SCD1* was found in lysoPE 18:2-supplemented cells. The decrease in *PPARγ* and *CD36* levels suggests a protective response against hepatic steatosis (Figure 8).

## 5. Conclusions

In this study, we demonstrated that lysoPE supplementation induced lipid droplet formation in a human liver-derived cell line, and revealed that lysoPE induced increases in TAG, CE, PE, lysoPE and PC, containing linoleic acyl. Further, we found that lysoPE supplementation led to the downregulation of *ATGL* and TAG accumulation, which may be involved in impaired lipid droplet catabolism. According to these findings, lysoPE might be a bioactive lipid that induces fatty liver formation. In that case, a protective response against hepatic steatosis might occur due to the decrease of gene expressions related to de novo lipogenesis and FA uptake. In terms of the limitations in this study, it should be noted that whether other lysoPE species possess the same effects remains unclear, as only lysoPE 18:2 was investigated in the present study. Additionally, since this experiment was based on cultured cells, it was difficult to elucidate whether the characteristics of lysoPE 18:2-supplemented cells were close to NAFL or NASH. Thus, the in vivo effects of lysoPE are worthy of investigation in order to clarify whether lysoPE plays a pathological role in the induction of fatty liver in animals and humans. Nevertheless, our data suggest that lysoPE could be established as a novel pharmacological target for hepatic steatosis.

## Data Availability

Raw data in this study are available from the corresponding author upon reasonable request.

## Supplementary Materials

The following supporting information can be downloaded at: https://www.mdpi.com/article/10.3390/nu14030579/s1. Table S1: Primer sequences of each gene used for real-time PCR. Scheme S1: Graphical scheme of the study approach. It is important to understand if lysoPE is involved in the alteration of lipid metabolism, including hepatic lipid accumulation, as the physiological functions of lysoPE have not yet been elucidated. In the present study, we focused on the physiological functions of lysoPE 18:2 and investigated whether it affected lipid accumulation in a human hepatoma cell line, C3A. First, we investigated the cellular toxicity of lysoPE 18:2 to determine the optimal concentration of lysoPE added. Second, we observed lipid droplet formation in the C3A cells supplemented with lysoPE 18:2 by using Oil Red O staining. Third, we investigated the intracellular lipid profile using LC-MS/MS to understand the biological effects of lysoPE. Last, we analyzed the expression of genes related to lipid metabolism and catabolism using real-time PCR.
