# Supplementary material for: Lysophosphatidylethanolamine Affects Lipid Accumulation and Metabolism in a Human Liver-Derived Cell Line
Source: Nutrients. 2022 Jan 28;14(3):579. doi: 10.3390/nu14030579 (PMC8839386; doi:10.3390/nu14030579)
Supplement: Supplementary file 1 [file nutrients-14-00579-s001.zip › Scheme S1.pdf]

Lysophosphatidylethanolamine affects lipid accumulation and metabolism in a human liver-derived cell line

Yusuke Yamamoto[1,†], Toshihiro Sakurai[1,†], Zhen Chen[1], Nao Inoue[1], Hitoshi Chiba[2] and Shu-Ping Hui[1,*]

1. Faculty of Health Sciences, Hokkaido University, Kita-12, Nishi-5, Kita-ku, Sapporo 060-0812, Japan; yammasxy@gmail.com (Y.Y.); sakura@hs.hokudai.ac.jp (T.S.); chenzhen@hs.hokudai.ac.jp (Z.C.); naobee0628@eis.hokudai.ac.jp (I.N.)
2. Department of Nutrition, Sapporo University of Health Sciences, Nakanuma Nishi-4-2-1-15, Higashi-ku, Sapporo 070-0894, Japan; chiba-h@sapporo-hokeniryou-u.ac.jp

* Correspondence: keino@hs.hokudai.ac.jp; Tel. & Fax: +81-11-706-3693

† These authors contributed equally to this work.

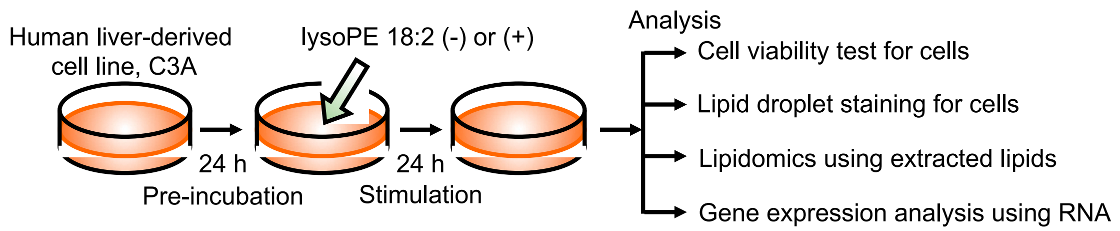

Scheme S1. Graphical scheme of the study approach. It is important to understand if lysoPE is involved in the alteration of lipid metabolism, including hepatic lipid accumulation, as the physiological functions of lysoPE have not yet been elucidated. In the present study, we focused on the physiological functions of lysoPE 18:2 and investigated whether it affected lipid accumulation in a human hepatoma cell line, C3A. First, we investigated the cellular toxicity of lysoPE 18:2 to determine the optimal concentration of lysoPE added. Second, we observed lipid droplet formation in the C3A cells supplemented with lysoPE 18:2 by using Oil Red O staining. Third, we investigated the intracellular lipid profile using LC-MS/MS to understand the biological effects of lysoPE. Last, we analyzed the expression of genes related to lipid metabolism and catabolism using real-time PCR.
