# Supplementary material for: Lysophosphatidylethanolamine Affects Lipid Accumulation and Metabolism in a Human Liver-Derived Cell Line
Source: Nutrients. 2022 Jan 28;14(3):579. doi: 10.3390/nu14030579 (PMC8839386; doi:10.3390/nu14030579)
Supplement: Supplementary file 1 [file nutrients-14-00579-s001.zip › Table S1.pdf]

Lysophosphatidylethanolamine affects lipid accumulation and metabolism in a human liver-derived cell line

Yusuke Yamamoto[1, †], Toshihiro Sakurai[1, †], Zhen Chen[1], Nao Inoue[1], Hitoshi Chiba[2] and Shu-Ping Hui[1,*]

1. Faculty of Health Sciences, Hokkaido University, Kita-12, Nishi-5, Kita-ku, Sapporo 060-0812, Japan; yammasxy@gmail.com (Y.Y.); sakura@hs.hokudai.ac.jp (T.S.); chenzhen@hs.hokudai.ac.jp (Z.C.); naobee0628@eis.hokudai.ac.jp (I.N.)
2. Department of Nutrition, Sapporo University of Health Sciences, Nakanuma Nishi-4-2-1-15, Higashi-ku, Sapporo 070-0894, Japan; chiba-h@sapporo-hokeniryou-u.ac.jp

* Correspondence: keino@hs.hokudai.ac.jp; Tel. & Fax: +81-11-706-3693

† These authors contributed equally to this work.

Table S1. Primer sequences of each gene used for real-time PCR

Gene	Species	Primer sequence (5'-3')
GAPDH	Human	F: GAAGGTGAAGGTCGGAGTC<br>R: GAAGATGGTGATGGGATTTC
ATGL	Human	F: ACCAGCATCCAGTTCAACCT<br>R: ATCCCTGCTTGCACATCTCT
DGAT1	Human	F: TATTGCGGCCAATGTCTTTGC<br>R: CACTGGAGTGATAGACTCAACCA
SCD1	Human	F: GACGATGAGCTCCTGCTGTT<br>R: CTCTGCTACACTTGGGAGCC
SREBP1	Human	F: CAGCCCACTTCATCAAGG<br>R: ACTGTTGCCAAGATGGTTCCG
PPARG	Human	F: CTTATCTATGACAGATGTGATCTT<br>R: GCTGGTCGATATCACTGGAGA
CD36	Human	F: GGCTGTGACCGGAACTGTG<br>R: AGGTCTCCAACTGGCATTAGAA

F: Forward, R: Reverse.
